# Supplementary material for: APOE4–Aβ synergy drives brain network dysfunction and neuronal lysosomal-ER proteostasis dysregulation a preclinical Alzheimer’s disease model
Source: bioRxiv. 2025 Nov 11:2025.11.11.687887. Preprint. [Version 1] doi: 10.1101/2025.11.11.687887 (PMC12642224; doi:10.1101/2025.11.11.687887)
Supplement: Supplement 1 [file NIHPP2025.11.11.687887v1-supplement-1.pdf]

bioRxiv preprint doi: https://doi.org/10.1101/2025.11.11.687887; this version posted November 12, 2025. The copyright holder for this preprint (which was not certified by peer review) is the author/funder, who has granted bioRxiv a license to display the preprint in perpetuity. It is made available under aCC-BY 4.0 International license.



## SUPPLEMENTAL FIGURES

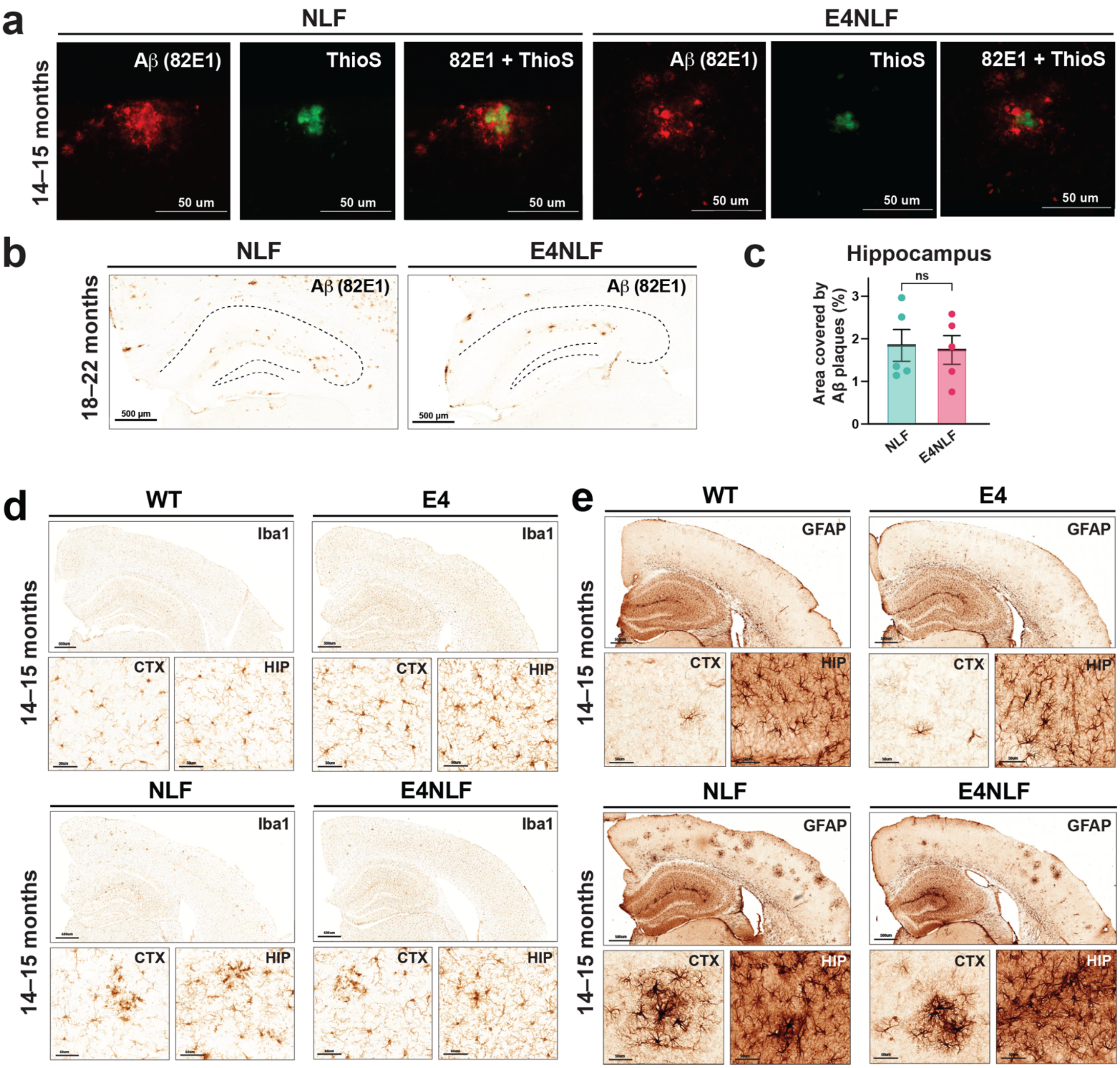


**Supplemental Fig. 1 (Related to Fig. 1): APOE4- and Aβ-dependent pathological changes in aged E4NLF mice.** Aged 14–15-month-old (a, d, and e) and 18–22-month-old (b, a) and WT, APOE4/E4 (E4), *App*$^{NLF/NLF}$, and APOE4/E4:*App*$^{NLF/NLF}$ (E4NLF) littermate mice were assessed by histological analyses.

bioRxiv preprint doi: https://doi.org/10.1101/2025.11.11.687887; this version posted November 12, 2025. The copyright holder for this preprint (which was not certified by peer review) is the author/funder, who has granted bioRxiv a license to display the preprint in perpetuity. It is made available under aCC-BY 4.0 International license.



(**a**) Representative hippocampal images from 14–15-month-old NLF and E4NLF mice stained with the human-specific Aβ antibody 82E1 (red) and Thioflavin-S (ThioS; green). Mature Aβ plaques in NLF and E4NLF mice display a Thioflavin-S-positive core.

(**b**) Representative hippocampal images of Aβ 82E1-immunostained from 18–22-month-old NLF and E4NLF mice.

(**c**) Quantification of hippocampal Aβ plaque burden expressed as the percent area covered by 82E1-positive staining in 18–22-month-old NLF and E4NLF mice. No significant differences were observed between 18–22-month-old NLF and E4NLF mice (unpaired two-tailed t-test; ns). Bars represent mean ± SEM.

(**d**) Representative hippocampal sections immunostained for Iba1 in 14–15-month-old WT, E4, NLF, and E4NLF mice. Quantification is shown in Fig. 1E.

(**e**) Representative hippocampal sections immunostained for GFAP in 14–15-month-old WT, E4, NLF, and E4NLF mice. Quantification is shown in Fig. 1E.

bioRxiv preprint doi: https://doi.org/10.1101/2025.11.11.687887; this version posted November 12, 2025. The copyright holder for this preprint (which was not certified by peer review) is the author/funder, who has granted bioRxiv a license to display the preprint in perpetuity. It is made available under aCC-BY 4.0 International license.



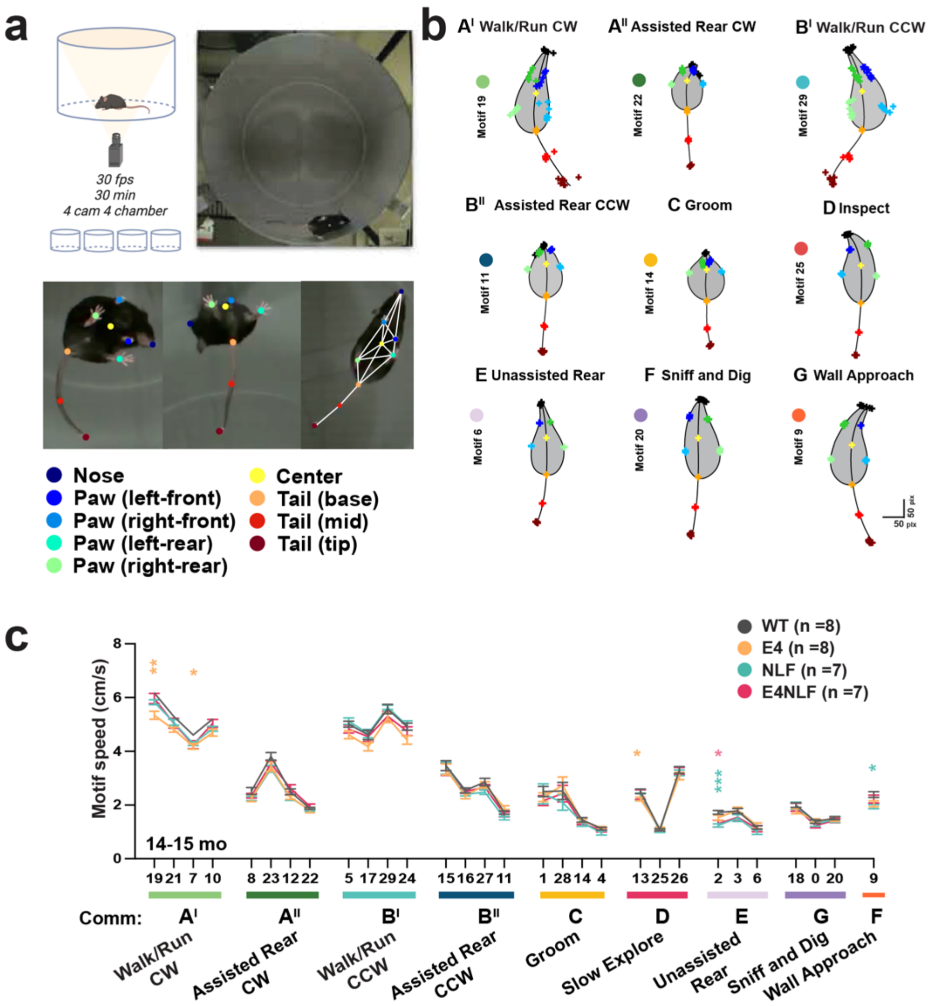


**Supplemental Fig. 2 (Related to Fig. 2): VAME-based unsupervised behavioral segmentation and motif characterization in the open field.** Aged (14–15-month-old) WT, E4, NLF, and E4NLF littermate mice were tested using the machine-learning VAME framework to assess spontaneous behavioral alterations.

(**a**) Experimental setup and pose-tracking pipeline. Mice were recorded for 30 min in a 4-camera circular open-field arena at 30 fps. Top: schematic of the 4-camera acquisition system and example overhead frame. Bottom: DeepLabCut-based pose estimation using 12 anatomical keypoints (nose, left/right front paws, left/right hind paws, body center, tail base, tail mid, and tail tip) used as inputs for variational animal motion embedding (VAME). Example trajectories and skeletal reconstructions illustrate pose-tracking precision during spontaneous behavior.

(**b**) Representative VAME-derived behavioral motifs identified from unsupervised clustering of pose-trajectory embeddings. Each schematic depicts the characteristic posture dynamics and centroid trajectory associated with a specific motif. We identified the following communities:

bioRxiv preprint doi: https://doi.org/10.1101/2025.11.11.687887; this version posted November 12, 2025. The copyright holder for this preprint (which was not certified by peer review) is the author/funder, who has granted bioRxiv a license to display the preprint in perpetuity. It is made available under aCC-BY 4.0 International license.



**A′** Walk/Run clockwise (CW): sustained forward locomotion with coordinated paw placement.

**A″** Assisted Rear CW: rear posture supported by the wall with clockwise body rotation.

**B′** Walk/Run counterclockwise (CCW): robust locomotion with CCW trajectory curvature.

**B″** Assisted Rear CCW: rear posture supported by the wall with CCW rotation.

**C** Groom: cyclical head–body movements characteristic of self-grooming.

**D** Inspect: stationary exploratory posture with forward head extension.

**E** Unassisted Rear: vertical rearing without wall support.

**F** Sniff and Dig: nose-down exploratory sequences with paw and snout movements.

**G** Wall Approach: directed movement toward the arena perimeter followed by orienting behavior.

Scale bar, 50 px (approx.).

(**c**) Motif-specific speed across genotypes. Line plots show average centroid speed (cm/s) for each motif, aligned to the corresponding motifs and communities. Motif speeds were comparable across genotypes, with modest differences limited to a subset of high-speed locomotor motifs (two-way ANOVA with Bonferroni post hoc tests; $p < 0.05$ where indicated). Data represent mean ± SEM.

bioRxiv preprint doi: https://doi.org/10.1101/2025.11.11.687887; this version posted November 12, 2025. The copyright holder for this preprint (which was not certified by peer review) is the author/funder, who has granted bioRxiv a license to display the preprint in perpetuity. It is made available under aCC-BY 4.0 International license.



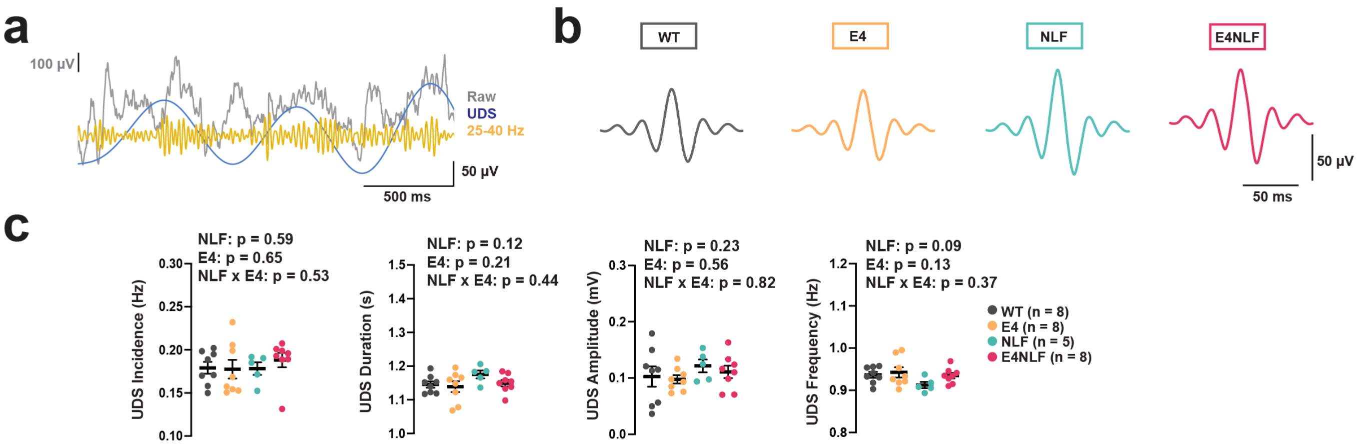


**Supplemental Fig. 3 (Related to Fig. 3): Quantification of cortical Up–Down state (UDS) dynamics across genotypes.** Aged (18–22-month-old) WT, E4, NLF, and E4NLF littermate mice (WT n = 8, E4 n = 8, NLF n = 5, E4NLF n = 8) were implanted in the posterior parietal cortex with wireless EEG/EMG transmitters and recorded for two weeks to assess slow-oscillation dynamics during NREM sleep.

(**a**) Representative raw EEG trace (grey) with band-pass filtered 25–40 Hz envelope (yellow) illustrating alternating Up and Down states detected during NREM sleep. Blue line shows the algorithm-derived UDS segmentation.

(**b**) Average Up–Down state waveforms across genotypes, aligned to Up-state onset. WT (black) displays a canonical depolarizing Up state followed by a hyperpolarizing Down state; E4 (orange), NLF (green), and E4NLF (magenta) mice show comparable waveform morphology.

(**c**) Quantification of UDS parameters, including incidence (Hz), duration (s), amplitude (µV), and oscillation frequency (Hz). Across all measures, no significant APOE4-, NLF-, or APOE4×NLF-interaction effects were detected (two-way ANOVA with NLF and E4 as factors; indicated p values). Scatter plots show individual mice and group means ± SEM.
